# High-grade dysplastic spondylolisthesis: surgical technique and case series

**DOI:** 10.1007/s12306-022-00763-w

**Published:** 2022-10-01

**Authors:** C. Faldini, F. Barile, M. Ialuna, M. Manzetti, G. Viroli, F. Vita, M. Traversari, A. Rinaldi, T. Cerasoli, A. Paolucci, G. D’Antonio, A. Ruffilli

**Affiliations:** IRCCS Istituto Ortopedico Rizzoli, 1st Orthopaedics and Traumatology Clinic, University of Bologna, Bologna, Italy

**Keywords:** Spondylolisthesis, All posterior-one stage surgical technique, Reduction and fusion, Lumbosacral angle, Slip angle

## Abstract

**Purpose:**

The aim of the present study is to evaluate the results of our all posterior-one stage surgical technique for the reduction and fusion of high-grade high-dysplastic spondylolisthesis.

**Methods:**

Patients over 11 years old with high-grade spondylolisthesis treated by reduction and circumferential fusion with a posterior-only approach were reviewed. Data about operative time, blood loss, length of stay, intra- and postoperative complications were collected. Meyerding grade (M), lumbar lordosis (LL), thoracic kyphosis (TK), pelvic incidence (PI), pelvic tilt (PT), lumbosacral angle (LSA), slip angle (SLIP), lumbar index (LI) and severity index were measured on preoperative and last follow-up. Sagittal vertical axis (SVA) was used to assess sagittal balance.

**Results:**

Of the 14 included patients, L5-S1 arthrodesis was performed in 12 cases, and L4-S1 was performed in 2 cases. Average surgical time was 275 ± 65 min; average blood loss was 635 ± 375 mL. Average length of stay of was 3.9 ± 1.5 days. The SLIP angle improves from 33.8° ± 7.3° to 6.4° ± 2.5°, (*p* = 0.002); the lumbosacral angle improves from 68.8° ± 18.6° to 100.7° ± 13.2°, (*p* = 0.01); and the SVA decreased from 49.4 ± 22.1 mm to 34.4 ± 8.6 mm (*p* = 0.02). No significant changes were observed in PI, PT and SS. Thoracic kyphosis (TK) and lumbar lordosis (LL) did not change significantly. At last follow-up, no patient had surgical site infection or mechanical complications; no pseudoarthrosis was observed. No revision surgery was performed.

**Conclusion:**

Although technically demanding, reduction and fusion with one stage all posterior approach prove to be a safe and effective.

## Introduction

**Fig. 1:**
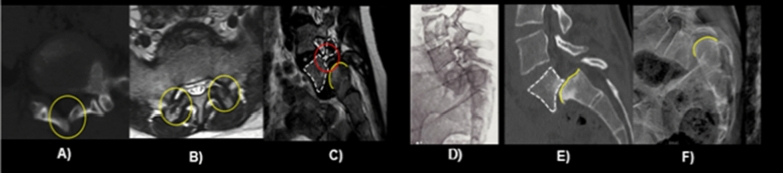
**A** schisis of L5 and S1 laminae, **B** sagittal orientation of articular facets, **C** L5 pedicle hypoplasia, **D** slippage of the L5 vertebral body, **E** trapezoidal L5 body, **F** rounded sacral dome

High-dysplastic spondylolisthesis is a rare entity that still represents a very controversial topic in spine surgery. In fact, while the distinction between high- and low-grade spondylolisthesis is well established as an anterior translation greater than 50% according to the Meyerding Classification [1], there is no consensus to distinguish between low and high-dysplastic cases. The most practical classification system in terms of prognosis and therapy is Marchetti and Bartolozzi’s [2]. This classification divides spondylolisthesis into two groups, developmental or acquired. Developmental spondylolisthesis is further divided into two types, both with lysis and elongation: low dysplastic and high dysplastic, depending on the severity of the bony dysplastic changes present on the L5 and S1 vertebrae and on the risk of slip progression. The low-dysplastic type is described as having a relatively normal lumbosacral profile, a normal rectangular L5 body, a normal S1 superior endplate, no pelvic retroversion and very low risk of slip progression. Conversely, high-dysplastic spondylolisthesis is characterized by congenital dysplasia of the posterior elements of the lumbosacral junction; in a skeletally immature patients, when vertical position is reached, these abnormalities lead to lysis or elongation of the pars interarticularis, slippage and trapezoidal deformation of L5 and a dome-shaped superior endplate of S1 (Fig. 1).

The progressive deformation of osseous and ligamentous structures promotes slippage and leads to kyphotic deformity of the lumbosacral junction. High-dysplastic spondylolisthesis is extremely progressive, and surgery is almost invariably needed. Therefore, distinguishing between low- and high-dysplastic cases is of foremost importance in order to choose the best course of treatment. Marchetti and Bartolozzi did not identify strict criteria to differentiate these two subtypes. For this reason, Lamartina et al. [3] introduced the severity index (SI). As demonstrated by Vidal and Marnay [4], SI is < 20% in normal subjects and in low-dysplastic spondylolisthesis since there is no pelvic retroversion, while it is > 20% in high-dysplastic spondylolisthesis.

Surgical treatment of patients affected by high-grade high-dysplastic spondylolisthesis still represents a challenge for spine surgeons. The importance of correcting the deformity by reducing slippage has been clear since the 1970s, when Scaglietti developed a non-surgical reduction technique with plaster cast [5]. Some years later, thanks to Vidal and Marnay [4], the knowledge of the pathological anatomy of this disease became deeper and four fundamental components have been identified: slippage, pelvic retroversion, verticalization of the sacrum and forward displacement of the hips. These factors cause the loss of normal sagittal alignment, and all of them need to be addressed to obtain a clinical and functional satisfactory result. The best surgical technique that combines efficacy in the deformity correction and safety in terms of neurological risks has not yet been established.

The aim of the present study is to evaluate the results of our all posterior-one stage surgical technique for the reduction and fusion of high-grade high-dysplastic spondylolisthesis in a homogeneous cohort of patients.

## Materials and methods

### Study sample

A retrospective review of patients over 11 years old with high-grade spondylolisthesis treated by reduction and circumferential fusion with a posterior-only approach was performed.

Inclusion criteria were as follows: a diagnosis of high-grade spondylolisthesis (confirmed by two experienced spine surgeons who reviewed the X-rays), grade 3 or 4 according to Meyerding classification [1] treatment with posterior-only reduction and circumferential fusion, a minimum 2-year follow-up.

Included patients were evaluated preoperatively and at last follow-up (minimum 2 years).

### Data collection

Medical records of included patients were reviewed and analysed. Data about operative time, blood loss, length of stay, intra- and postoperative complications were collected.

Meyerding grade (M), lumbar lordosis (LL), thoracic kyphosis (TK), pelvic incidence (PI), pelvic tilt (PT) [6], lumbosacral angle (LSA) [7], slip angle (SLIP) [8], lumbar index (LI)[9] and severity index [3] were measured on pre- and last follow-up full-length standing anteroposterior and lateral side radiographs. Sagittal vertical axis (SVA) was used to assess sagittal and coronal imbalance [6].

### Surgical planning and technique

**Fig. 2:**
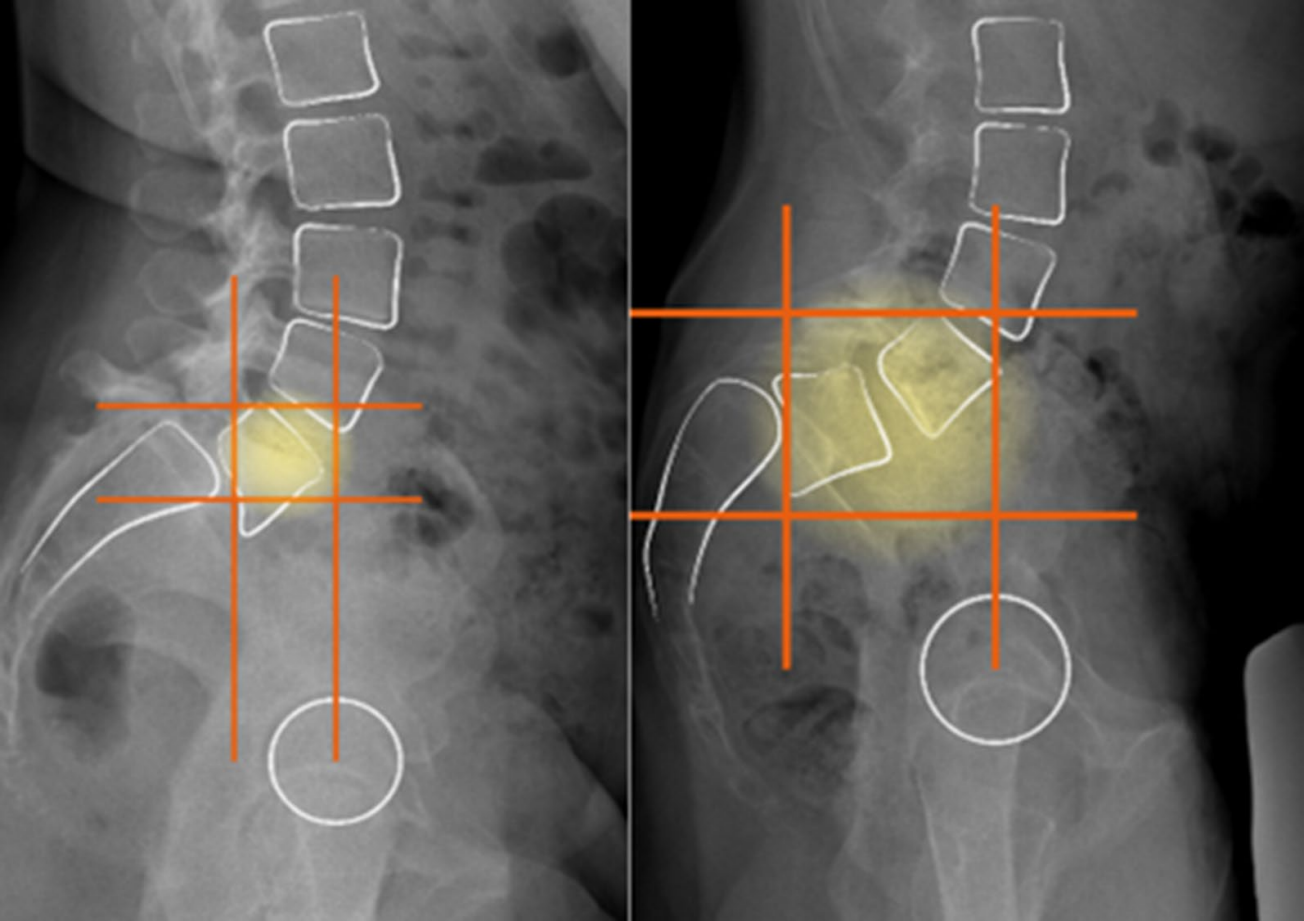
Two example of fusion area preoperatively defined by identifying the unstable zone according to Lamartina et al. [3]. Left: only L5 was included. Right: L4-L5 was included

A surgical planning was performed on preoperative full-length standing anteroposterior and lateral side radiographs. The fusion area was preoperatively defined identifying the unstable zone according to Lamartina et al. [3] (Fig. 2); therefore, L4 was included in the fusion area when it laid in the Marnay’s square [4].

**Fig. 3:**
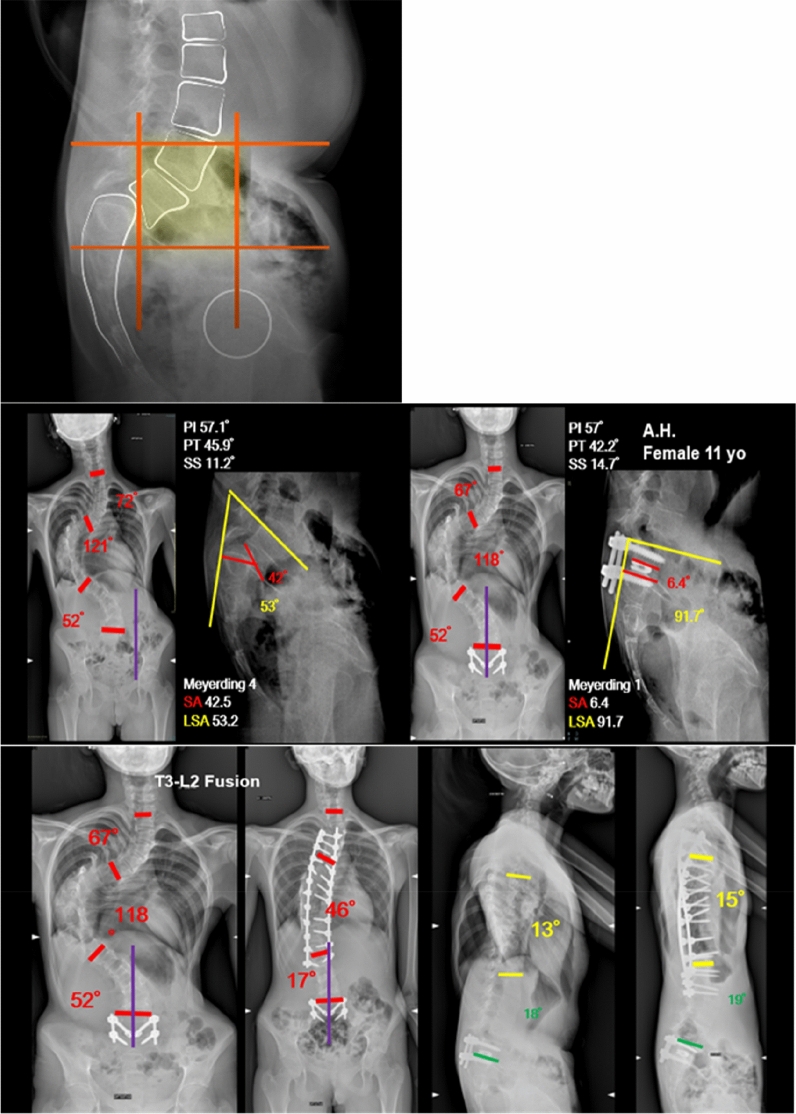
A case of spondylolisthesis associated with adolescent idiopathic scoliosis

When an associated thoracic scoliosis requiring future surgical treatment was present (2 cases), L4 was not included in the fusion area even if it laid in the unstable zone, in order to spare motion segments (Fig. 3).

Implanted screws were monoaxial on S1 and reduction screws on L5.

All surgeries were performed by the first author. After general anaesthesia, the patient was positioned prone on the Allen® table with somatosensory and motor evoked potentials (SSEPs and MEPs). Under fluoroscopic control, the L5 vertebra was localized.

Standard posterior midline approach with subperiosteal exposure was performed. Careful exposure of the posterior element is crucial, extending laterally to the transverse processes of L5 slipped vertebra.

**Fig. 4:**
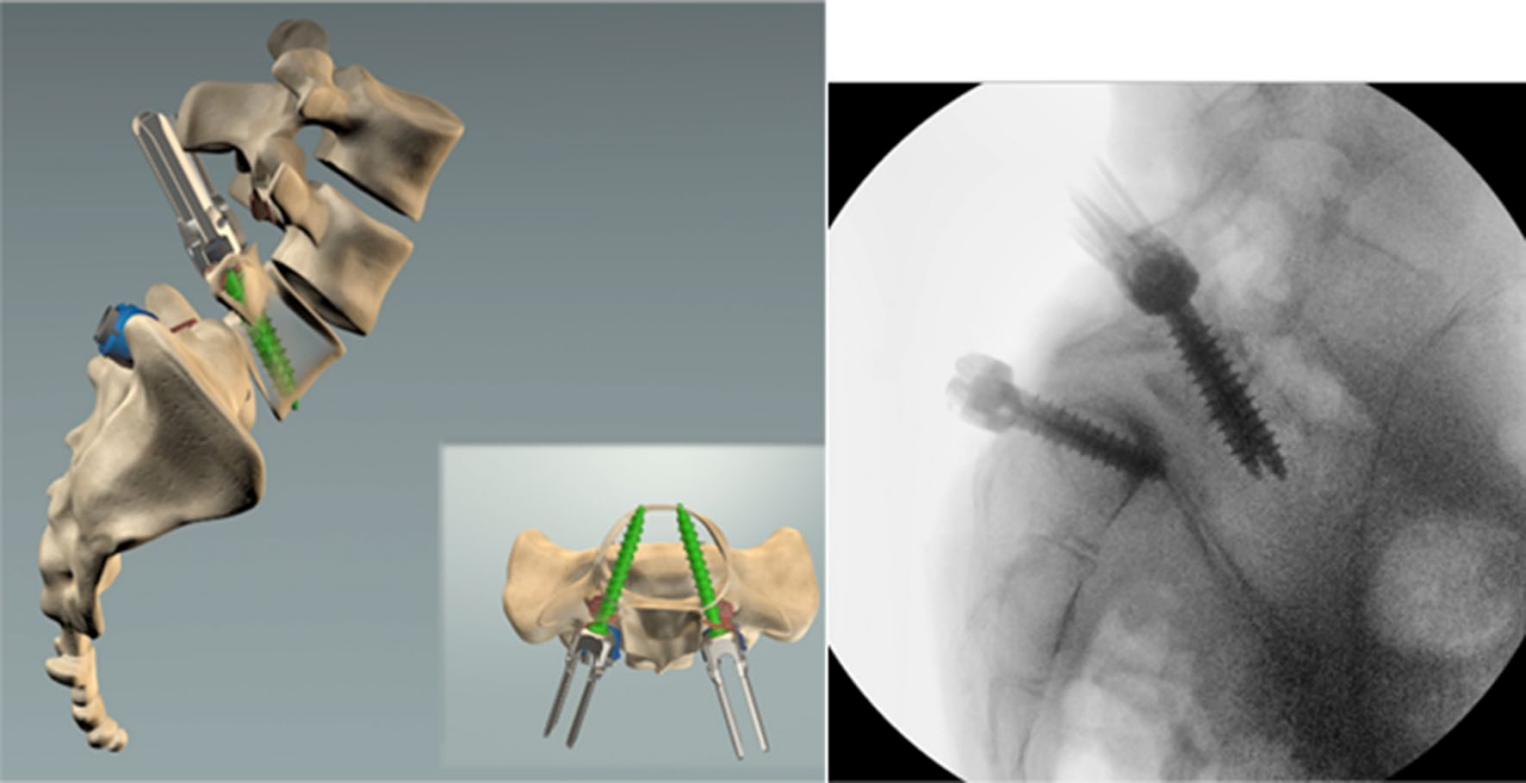
Left: 3D reconstruction. Right: position of the screw was checked by fluoroscope. It is crucial to adopt a far lateral entry point, following a funnel, convergent, trajectory on the axial plane with a bicortical purchase

Screws were placed with a free-hand, power-assisted technique [10]. In order to minimize the pull-out risk during the reduction manoeuvre, it is crucial to adopt a far lateral entry point, following a funnel, convergent, trajectory on the axial plane with a bicortical purchase. On the sagittal plane, the screws must follow an anatomic trajectory that allows to perform a compression manoeuvre for the correction of the lumbosacral kyphosis. It was important that the entry point was as more lateral as possible to maximize the convergence of the screw trajectory, further increasing the pull-out strength (Fig. 4).

**Fig. 5:**
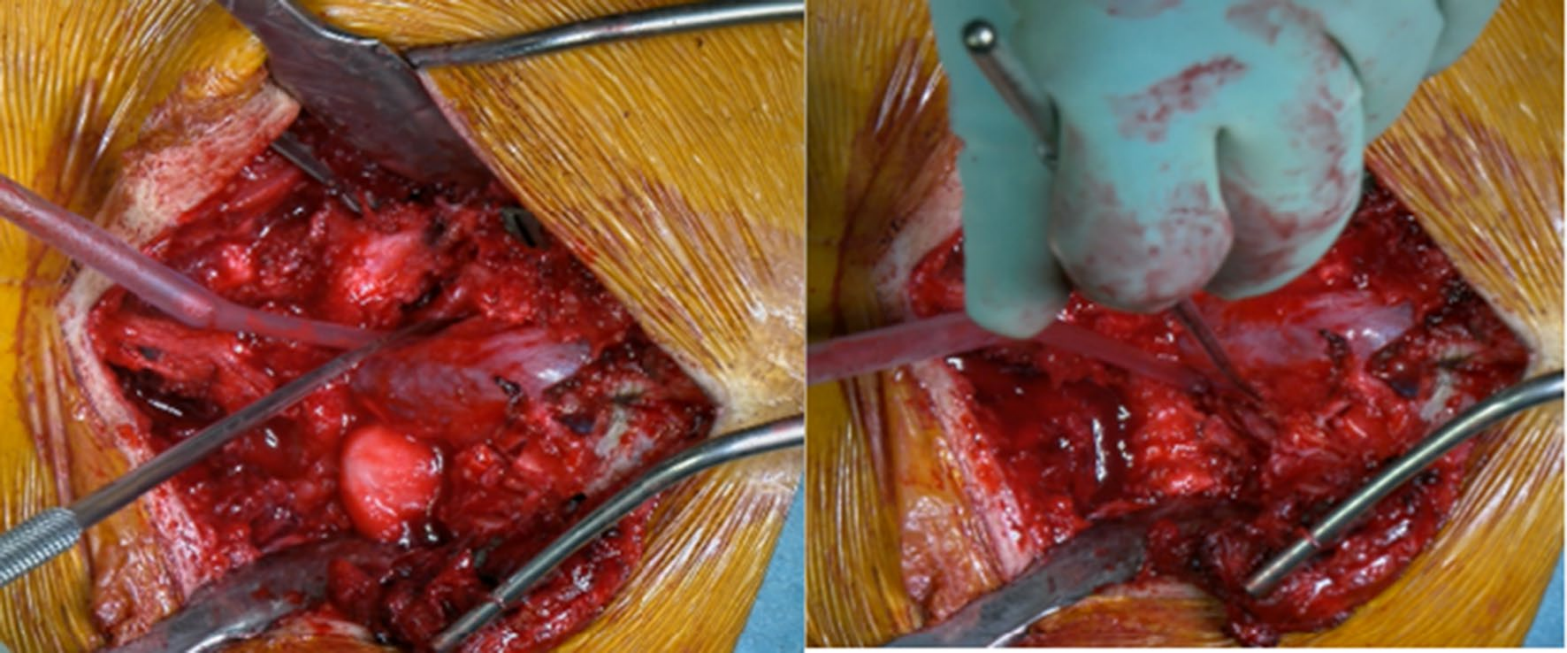
L5 roots were bilaterally exposed

Then, extended L5 laminectomy was performed; care should be taken to remove the inferior facets of L5 and the Gill’s fragment, extending laterally to completely expose the roots. This step is crucial to avoid roots traction during the reduction manoeuvre (Fig. 5).

After the laminectomy, an extensive release is necessary to allow a complete reduction of the deformity. Therefore, a careful discectomy was performed. The pathological anatomy of the disc needs to be kept in mind: in fact, in high-grade dysplastic spondylolisthesis the L5-S1 space is severely kyphotic.

The discectomy was performed bilaterally and far lateral under the L5 roots, to allow a complete mobilization of the slipped vertebra. In patients with a dome-shaped sacrum, a sacral dome resection osteotomy was performed: in fact, in these cases the reduction manoeuvre (that is not only translational but also rotational) and the consequent elongation of the lumbosacral junction put the patient at risk of neurological complications.

**Fig. 6:**
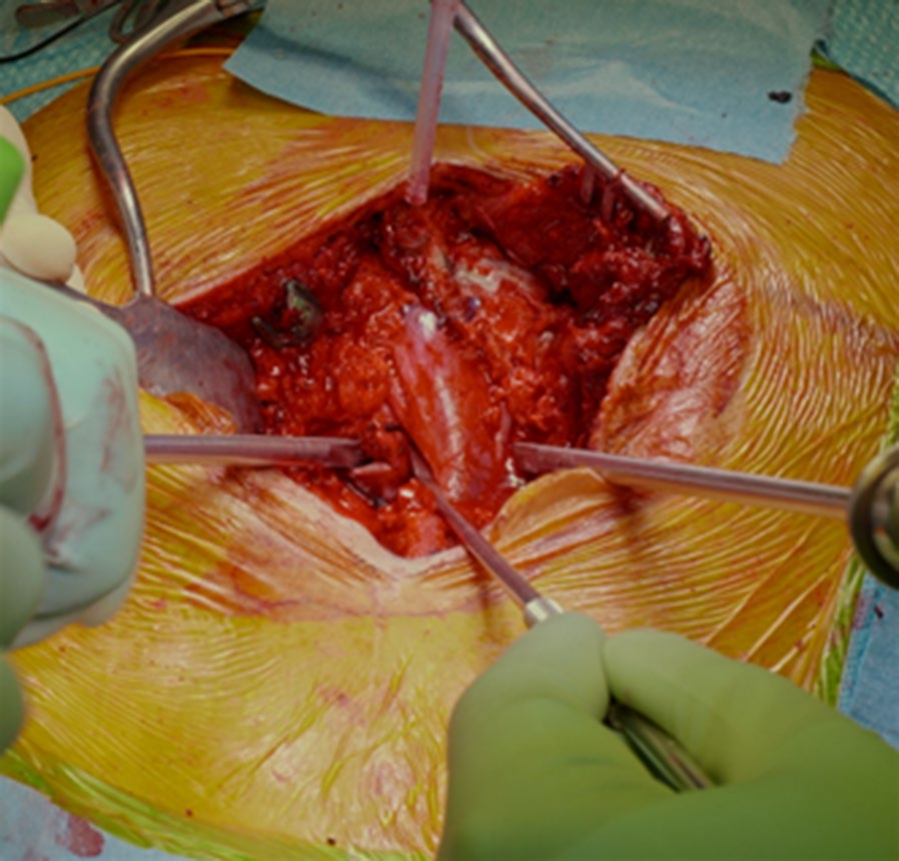
reamers-distractors were kept in place. It is important to note that the L5-S1 disc-space was kyphotic

Progressive reamers were used to remove all the discal tissue; the two final reamers-distractors were kept in place (Fig. 6).

**Fig. 7:**
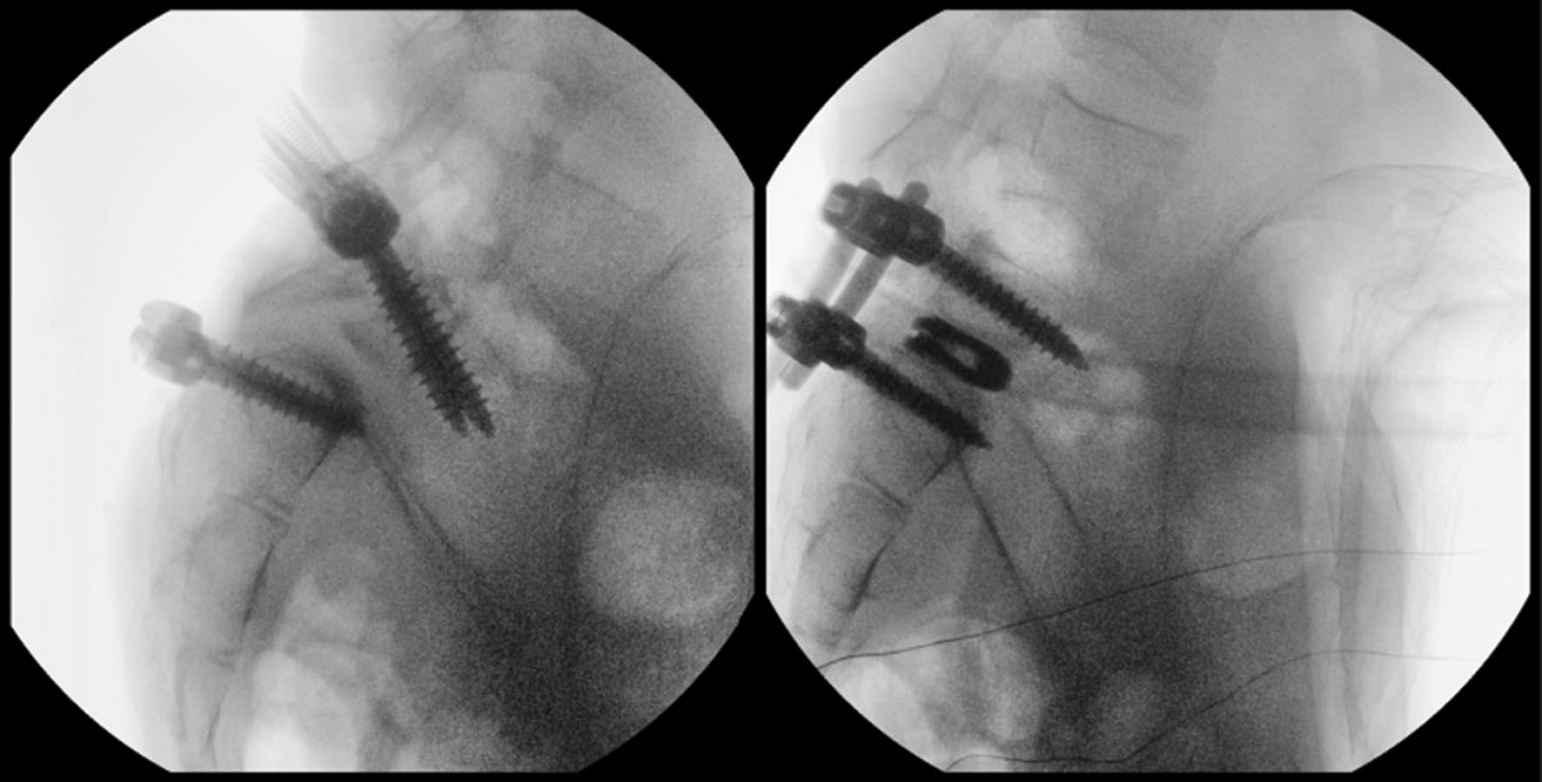
Final reduction is checked by fluoroscope

Then, two lordotic rods were simultaneously engaged in the S1 screws and two persuaders were placed on the L5 reduction screws and progressively tightened, while the reamers-distractors acted as levers to facilitate reduction. The manoeuvre was performed gradually, always paying attention to the L5 roots. Finally, two titanium cages were placed, and compression was performed to restore L5-S1 lordosis (Fig. 7).

Postoperatively, the patients started ambulation on the 2nd day, without any orthosis. Patients were advised to restrict forward bending for 1 month and to avoid contact sports for at least 3 months after surgery.

### Patients’ characteristics

Fourteen patients were included (2 males and 12 females), with a mean follow-up of 37 ± 14 (range 23–45) months. The average age was 25.1 ± 12.3 (range 12.2–44.5). All patients were affected by high-grade high-dysplastic spondylolisthesis (average severity index 56.6 ± 14.2%).

Patient’s characteristics are summarized in Table 1.

**Table 1:** Patient’s characteristics

	Patient’s characteristics
Patients n	14 (2 males, 12 females)
Average age (years)	25.1 ± 12.3 (range 12.2–44.5)
Meyerding grade	4(7) 3(7)
Average lumbar index (LI)	66.5% ± 11.9%
Average severity index	56.6 ± 14.2%
Average surgical time (mins)	275’ ± 65’
Average blood loss (mL)	635 mL ± 375 mL
Average length of stay (days)	3.9 ± 1.5 (range 3–6)
Average follow-up (months)	37 ± 14 (range 23–45)

N = number. Mins = minutes

### Statistical analysis

Parametric test was used to compare samples in case of continuous variables, normal distribution and appropriate numerousness. The Shapiro–Wilk test was used to verify normal distribution. The Levene test was used to evaluate homogeneity of the variances. As parametric test, we used two-tailed Student t test to compare the average of the variables for homoscedastic paired groups. As nonparametric test, we used the two-tailed Wilcoxon signed-rank test for paired group. Continuity correction was applied in case of discrete distribution. *P*-values < 0.05 were considered significant. SPSS 17.0 statistical analysis software (SPSS Inc., Chicago, Illinois, USA) was used to perform statistical analysis.

## Results

Of the 14 included patients, 7 had Meyerding grade 3 spondylolisthesis, and 7 had Meyerding 4. L5-S1 arthrodesis was performed in 12 cases; L4-S1 was performed in 2 cases.

Average surgical time was 275 ± 65 min, and average blood loss was 635 ± 375 mL (Table 1). Intraoperatively, the surgical planning was respected in all cases, with no need to extend the fusion area due to screws pull-out during corrective manoeuvre; no alteration of SSEPS and MEPS was registered. Patients were discharged after an average length of stay of was 3.9 ± 1.5 days.

Significant results were achieved in correcting the SLIP angle (from 33.8° ± 7.3° to 6.4° ± 2.5°, *p* = 0.002), the lumbosacral angle (from 68.8° ± 18.6° to 100.7° ± 13.2°, *p* = 0.01) and the SVA that decreased from an average 49.4 ± 22.1 mm preoperatively to an average 34.4 ± 8.6 mm postoperatively (*p* = 0.02).

Considering the spinopelvic parameters, no significant changes were observed in PI, PT and SS (Table 2).

**Table 2:** Preoperative and postoperative parameters. SLIP angle, Lumbosacral angle and SVA varied significantly (p < 0.05)

	Preoperative	Postoperative	*p* value
SLIP angle	33.8° ± 7.3°	6.4° ± 2.5°	**0.002**
Lumbosacral Angle (LSA)	68.8° ± 18.6°	100.7° ± 13.2°	**0.01**
PI	73.5 ± 11.2	73 ± 13.1	0.96
PT	31.8 ± 12.7	30.7 ± 9.8	0.88
SS	41.7 ± 18.1	42.3 ± 12.2	0.86
LL	50.8 ± 18.4	49.2 ± 18.7	0.81
TK	35.8 ± 12.3	39 ± 11.2	0.25
SVA (mm)	49.4 ± 22.1	34.4 ± 8.6	**0.02**

PI = pelvic incidence, PT = pelvic tilt, SS = sacral slope, LL = lumbar lordosis, TK = thoracic kyphosis, SVA = sagittal vertical axis

2. Preoperative and postoperative parameters. SLIP angle, lumbosacral angle and SVA varied significantly (*p* < 0.05). PI = pelvic incidence, PT = pelvic tilt, SS = sacral slope, LL = lumbar lordosis, TK = thoracic kyphosis, SVA = sagittal vertical axis.

Postoperatively, no patient had surgical site infection or mechanical complications. Two patients (14.2%) showed (despite uneventful neuromonitoring) L5 sensory symptoms, which regressed within the first week: this was probably due to the reaction of L5 roots to manipulation and tension.

None of the patients received revision surgery. At last follow-up, no clinical or radiographical sign of pseudoarthrosis was observed.

## Discussion

This is a retrospective analysis of 14 patients with high-grade spondylolisthesis treated by one-stage reduction and fusion from posterior-only approach. The presented technique, although technically demanding, proved to be effective and safe in correcting the deformity without mechanical or neurological complications.

The most important outcome of this study is the efficacy of the presented technique in restoring both local and global sagittal alignment: in fact, SLIP angle, lumbosacral angle and SVA all significantly improved. In particular, an impressive reduction of the SLIP angle (that decreased from 33.8° ± 7.3° to 6.4° ± 2.5°, *p* = 0.002) can be observed.

Nevertheless, spinopelvic parameters did not change significantly between pre- and postoperative. This is still a controversial topic; in fact, while it is clear that performing reduction surgery for spondylolisthesis leads to a partial or complete correction of translation and regional kyphosis, the effect of surgery on pelvic orientation remains uncertain. Only a few authors reported a statistically significant improvement of spinopelvic parameters [3, 11–20]; although the results reported by these authors are statistically significant because of the homogeneous direction of the values, their magnitude is modest and their clinical or functional importance has not yet been demonstrated. Moreover, as stated by Rivoiller et al. [21], the endpoint of a reduction procedure is restoration of global spinal balance through correction of the kyphosis at the lumbosacral junction; this goal has been achieved by the presented technique, with a complete normalization of the SVA (that decreased from 49.4 ± 22.1 mm to 34.4 ± 8.6 mm, *p* = 0.02).

As for safety, neurological complications represent a great concern when dealing with high-grade spondylolisthesis. Therefore, in situ fusion is preferred by some authors [22] to avoid L5 roots stretch [23, 24]. However, in the authors’ opinion, reduction of the deformity is of foremost importance to restore global and sagittal alignment [21, 25, 26]. The presented technique proved to be safe, with transient L5 sensory symptoms in 2 cases. A wide laminectomy, a careful discectomy and—when needed—a sacral dome osteotomy, are essential steps to obtain a low neurological complications rate.

In conclusion, although technically demanding, reduction and fusion with one stage all posterior approach prove to be a safe and effective technique to deal with high-grade spondylolisthesis. This technique restored good local and global alignment, without permanent neurological complications.

## Data Availability

The data that support the findings of this study are available from the corresponding author upon reasonable request.
