# Correction to: High-grade dysplastic spondylolisthesis: surgical technique and case series

**DOI:** 10.1007/s12306-022-00766-7

**Published:** 2022-11-09

**Authors:** C. Faldini, F. Barile, M. Ialuna, M. Manzetti, G. Viroli, F. Vita, M. Traversari, A. Rinaldi, T. Cerasoli, A. Paolucci, G. D’Antonio, A. Ruffilli

**Affiliations:** IRCCS Istituto Ortopedico Rizzoli, 1st Orthopaedics and Traumatology Clinic, University of Bologna, Bologna, Italy

## Correction to: MUSCULOSKELETAL SURGERY 10.1007/s12306-022-00763-w

The original version of this article unfortunately contained a mistake. The corrected details are given below for your reading.

Figure 2 caption should read as

Two example of fusion area preoperatively defined by identifying the unstable zone according to Lamartina et al. [3]. Left: only L5 was included. Right: L4-L5 was included

The Fig. 3 should have appeared as shown in the following page.

**Fig. 3:**
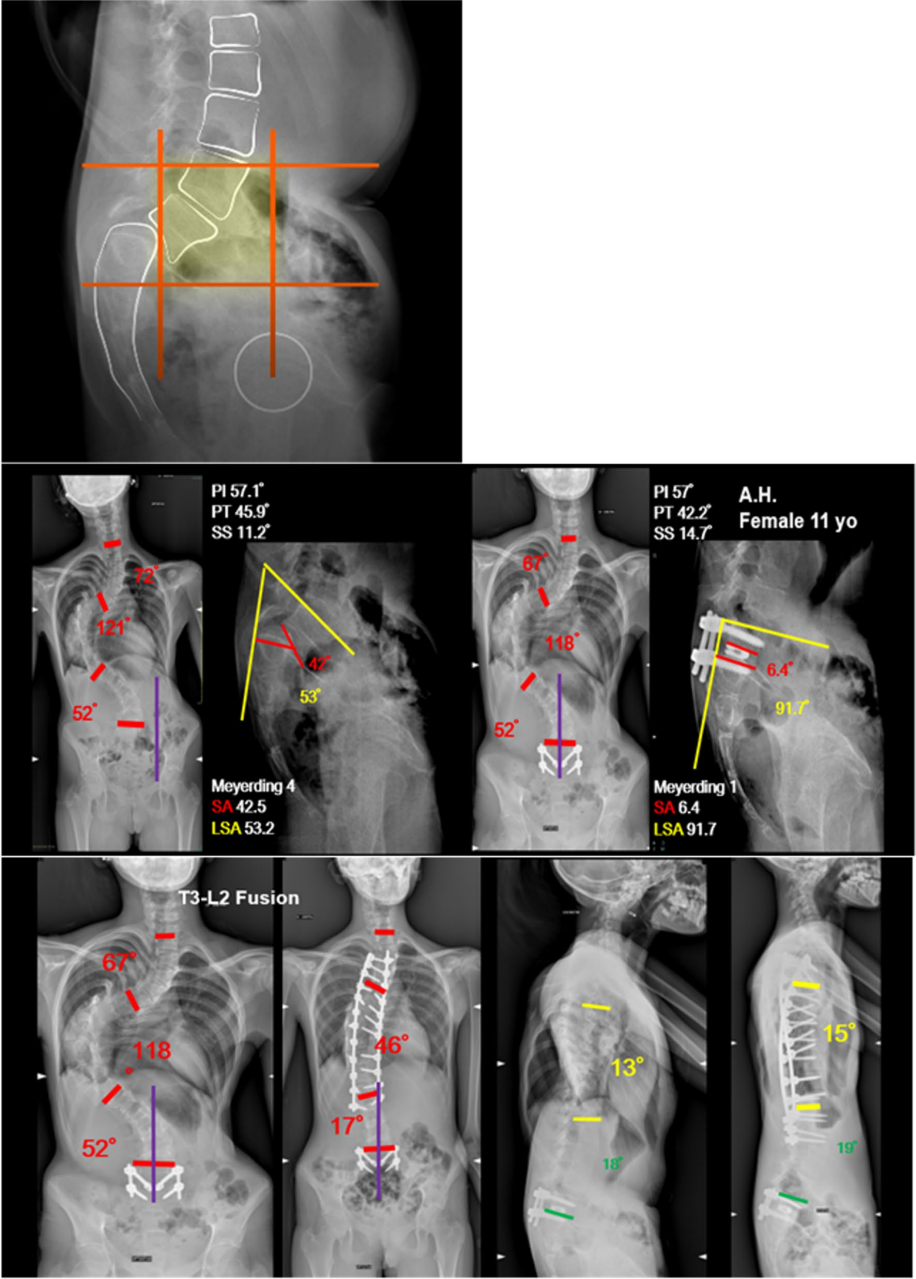
A case of spondylolisthesis associated with adolescent idiopathic scoliosis

Figure 7 caption should read as

Final reduction is checked by fluoroscope

The original article has been corrected.

